# Field drought conditions impact yield but not nutritional quality of the seed in common bean (*Phaseolus vulgaris* L.)

**DOI:** 10.1371/journal.pone.0217099

**Published:** 2019-06-06

**Authors:** Millicent R. Smith, Erik Veneklaas, Jose Polania, Idupulapati M. Rao, Stephen E. Beebe, Andrew Merchant

**Affiliations:** 1 Sydney Institute of Agriculture, School of Life and Environmental Sciences, Faculty of Science, The University of Sydney, Sydney, NSW, Australia; 2 School of Plant Biology, The University of Western Australia, Crawley, WA, Australia; 3 Centro Internacional de Agricultura Tropical (CIAT), Cali, Colombia; Wageningen University, NETHERLANDS

## Abstract

Drought substantially limits seed yield of common bean (*Phaseolus vulgaris* L.) in the tropics. Understanding the interaction of drought on yield and the nutrient concentration of the seed is vital in order to supply nutrition to the millions of consumers who rely on common bean as a staple crop. Nevertheless, the impact of drought on common bean for both yield and nutrient concentration has not yet been concurrently investigated in a field environment. Using 10 bred lines developed by CIAT and its partners for their improved adaptation to drought and phosphorus deficiency, this study characterised the impact of drought on yield and nutrient concentration for leaf and seed tissue of common bean grown in the field. Drought significantly reduced leaf area (by ~50%), harvest index (by ~60%), yield (by ~70%), seed weight (by ~25%) and enriched carbon isotope abundance (δ^13^C) in the seed. Within the soluble leaf fraction, drought significantly decreased the concentration of mineral nutrients and amino acids, whereas no negative effect on the concentration of nutrients and amino acids was detected within the seed. Genotypic variation in nutrient concentration in both the leaf and seed tissue was identified and should be explored further to identify traits that may confer tolerance to abiotic stress.

## Introduction

Drought imparts a major restriction to agricultural production affecting a wide range of crops via yield losses and crop failure [1]. The impacts of drought on crop production have been well studied [2] and vary based on drought type, intensity and duration [3, 4]. Grain legumes are often grown in areas where drought is a substantial risk [5].

Common bean (*Phaseolus vulgaris* L.) is typically cultivated by smallholder farmers using minimal inputs in developing nations of tropical regions [6]. As the most important grain legume for human consumption [7], it is estimated that 60% of common bean production occurs under the risk of intermittent or terminal drought stress [8]. These conditions cause yield losses of between 10–100% [9]. Substantial research efforts have been made to improve the resilience of common bean to drought (see, [8]). Crucially, selection for drought adaptation mechanisms in common bean lines has not reduced yields under favorable conditions, while simultaneously improving adaptation under other abiotic stress conditions such as phosphorus deficiency and heat [10]. This suggests that breeding activities have resulted in improvements to the overall efficiency of the lines and may have selected for factors that enhanced sink strength (see, [10, 11]).

Genetic improvement of common bean, while leading to well-filled seeds under severe drought, may have correspondingly reduced the nutrient concentration of the seed. This ‘dilution’ effect has previously been reported in wheat (see, [12]). Concurrently in response to drought, decrease in seed nutrient content likely occurs as a consequence of the following changes: reduced mineral nutrient uptake due to reductions in soil moisture, decreased mineralization and reductions in mass flow and nutrient diffusion within the soil [13], and compromised remobilisation from vegetative to reproductive tissues. Nevertheless, little research has focused on the impact of drought on the nutritional content of grain legume species. In this study we consider the nutrient concentration of the seed as including mineral nutrients, amino acids and sugars as expressed on a per weight and per seed basis. Nutrient concentration for common bean has been described previously [7] and is freely available through a database (see for example, [14]), however research has tended to focus on protein, iron and zinc concentrations and availability due to their relative importance in the diets of consumers. Under drought conditions the plant may increase production of osmolytes [15], vary the enzymes involved in the synthesis of starch [16] or accumulate compounds, such as phytic acid (myo-inositol hexaphosphoric acid) that are antagonistic to digestion limiting the bio-availability of certain nutrients [17]. Combined, these components could influence the overall concentration and availability of protein and mineral nutrients within the seed. Measuring the soluble carbohydrates, soluble amino acids and mineral nutrient concentrations allows us to consider if any ‘dilution’ may have occurred as a consequence of selections of lines that increased sink strength. Additionally, if representative of the seed, the soluble leaf fraction may offer an easily accessible, rapidly analysed pool to predict future yield or nutrient quality in breeding programs.

Field based trials allow us to investigate plant responses to the effects of stress conditions as close as possible to emulating typical cultivation of common bean. This is particularly important for the effects of drought. Field evaluation of drought stress is the favored option for breeding programs as the plants are affected by the timing and total water supply for the growing season [3] as well as the multiplicity of stress components that constitute drought conditions (see for example, [18]). Realistic crop spacing also allows for root exploration that mimics production conditions which is critical when considering the impacts of drought on yield and nutrient concentration of the seed.

Under field conditions, at the International Centre for Tropical Agriculture (CIAT), in Palmira, Colombia, we aimed to investigate the impact of terminal drought on yield quantity and nutrient concentration of seed in advanced common bean lines, and to determine if improvements to the resilience of common bean through selection for drought stress may have inadvertently reduced nutritional quality. Specifically, we address the following hypotheses: (1) drought stress, imposed through lack of irrigation reduces the yield quantity of all common bean lines; 2) genotypic differences in yield reductions will be observed as a consequence of drought stress; (3) δ^13^C value in both leaf and seed tissue will decrease to indicate the severity of drought stress; and (4) drought will lead to a reduction in concentration of nitrogen and other mineral nutrients, amino acids and sugars within the soluble components of both leaf and seed tissues.

## Results

### Yield

**Fig 1:**
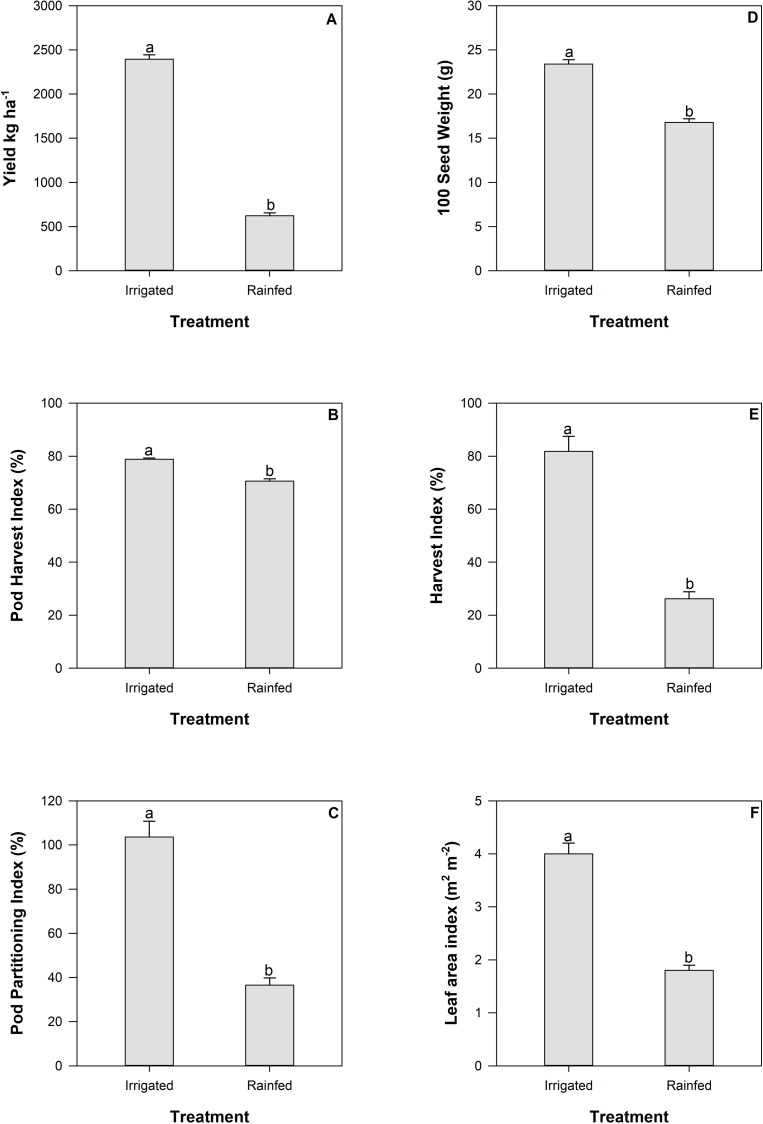
Yield (panel A), pod harvest index (panel B), pod partitioning index (panel C) seed weight (panel D), harvest index (panel E), and leaf area index (panel F) for common bean genotypes grown in the field at CIAT under irrigated or rainfed (drought) conditions. Multiple comparisons are denoted with lettering. Standard errors are shown where; n = 36.

**Fig 2:**
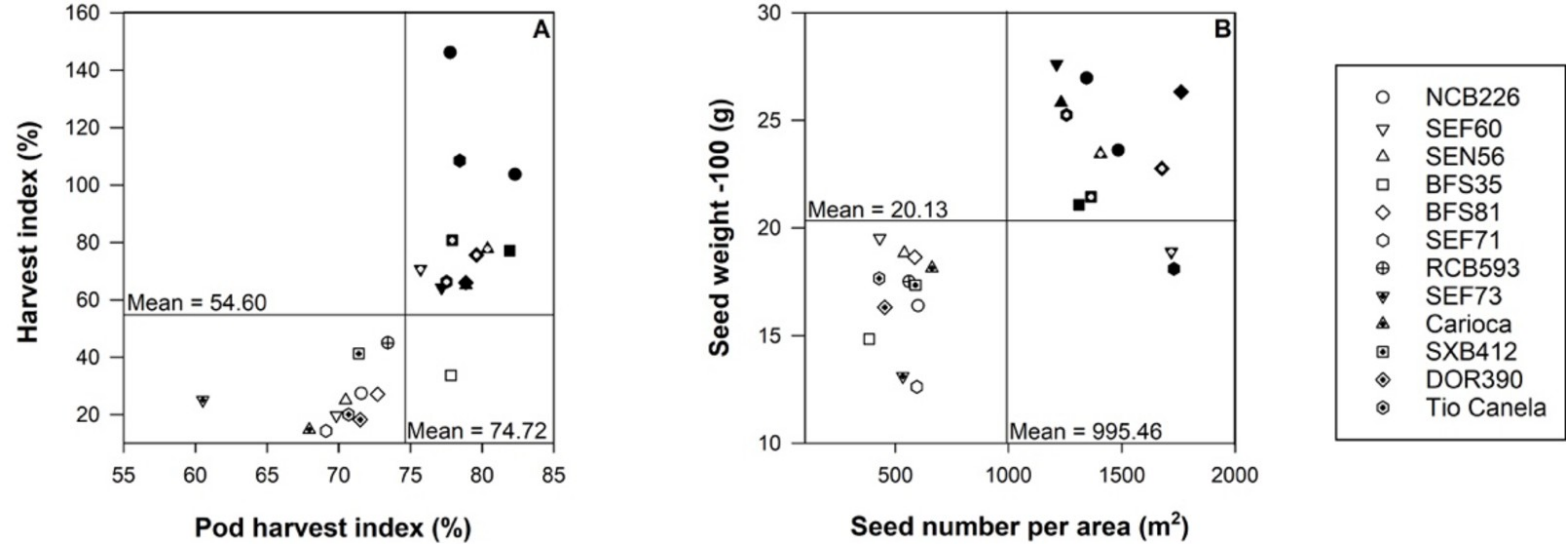
Harvest index (%) and pod harvest index (%) (panel A); seed weight -100 (g) and seed number per area (m^2^) (panel B) for 12 common bean genotypes (abbreviations centered over data points) grown in the field at CIAT under irrigated (blue) or rainfed (drought) (maroon) conditions. Standard errors have been removed for clarity (where n = 3).

Under irrigated and drought conditions days to flowering did not significantly differ (33.4±0.4 irrigated and 33.0±0.5 drought). Days to physiological maturity ranged from a mean of 61.5 days ±0.5 for all genotypes grown under irrigated conditions, compared to the shorter time period of 55.9 days ±0.8 for all genotypes grown under drought conditions. Significant differences were detected between irrigated and drought treatments (P < 0.001) with increases in yield (kg ha^-1^) (Fig 1A), pod harvest index (Fig 1B), pod partitioning index (Fig 1C), seed weight (Fig 1D) and harvest index (Fig1E) in response to irrigation (Fig 1). Harvest index under irrigated conditions was influenced by extra vegetative growth that occurred following sampling at mid-pod filling (see Eq 1 and Fig 1). Genotypic and treatment responses were found for each of these parameters excluding pod partitioning index which had no statistically significant genotypic response (data not shown). Differences in genotypic responses are displayed graphically as harvest index and pod harvest index (Fig 2A), seed weight and seed number (Fig 2B). For these parameters, genotypes bred for a combination of drought and low P tolerance (NCB226, SEF60, SEN56, BFS35, BFS81) along with those bred for adaptation to drought alone (SEF71, RCB593) typically demonstrated higher yield in comparison to the commercial checks (DOR390 and Tio Canela) (Fig 2).

### Patterns in carbon isotope abundance (δ^13^C)

**Fig 3:**
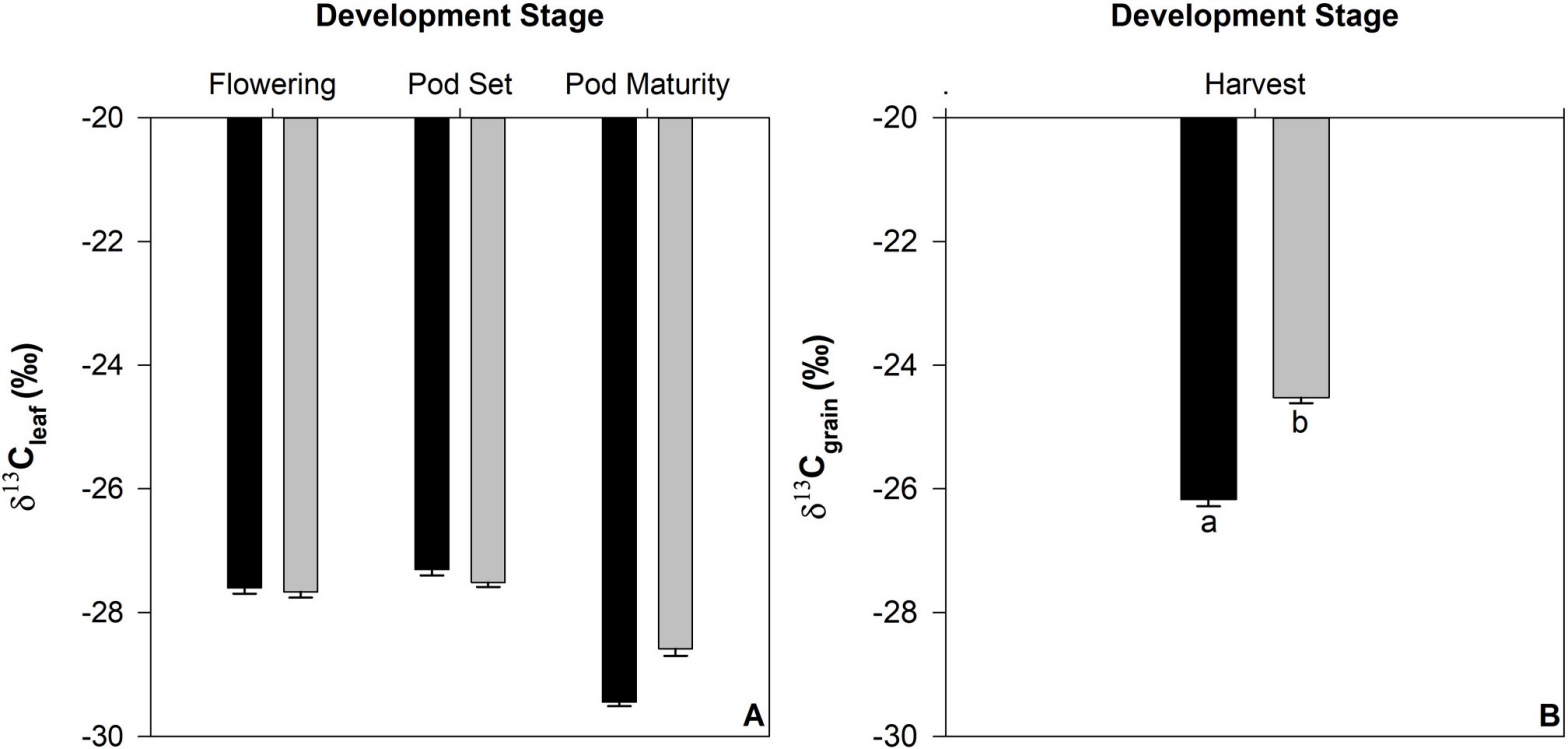
Carbon isotope abundance (δ^13^C) for leaf (panel A) and seed (panel B) tissue from 12 common bean genotypes grown in the field at CIAT under irrigated (black bars) or rainfed (drought) (grey bars) conditions over development for leaf tissue; flowering, pod set, pod maturity. Letters denote significance between treatments. Statistically significant differences were observed between each development stage (multiple comparisons are not shown). Standard errors are shown where; n = 36.

Carbon isotope abundance (δ^13^C) of the leaf tissue did not differ between irrigated and drought treatments in leaves collected at flowering or pod set (Fig 3A). δ^13^C of the leaf tissue decreased at pod maturity and statistically significant treatment differences were detected between irrigated and drought treatments (Fig 3A). No statistically significant genotypic variation of δ^13^C was detected for the leaf tissue (data not shown). δ^13^C for the irrigated seed tissue was significantly lower in comparison to the drought treatments (P < 0.001) (Fig 3B). No statistically significant genotypic variation of δ^13^C was detected for the seed tissue (data not shown).

### Impact of drought on nutrients

**Fig 4:**
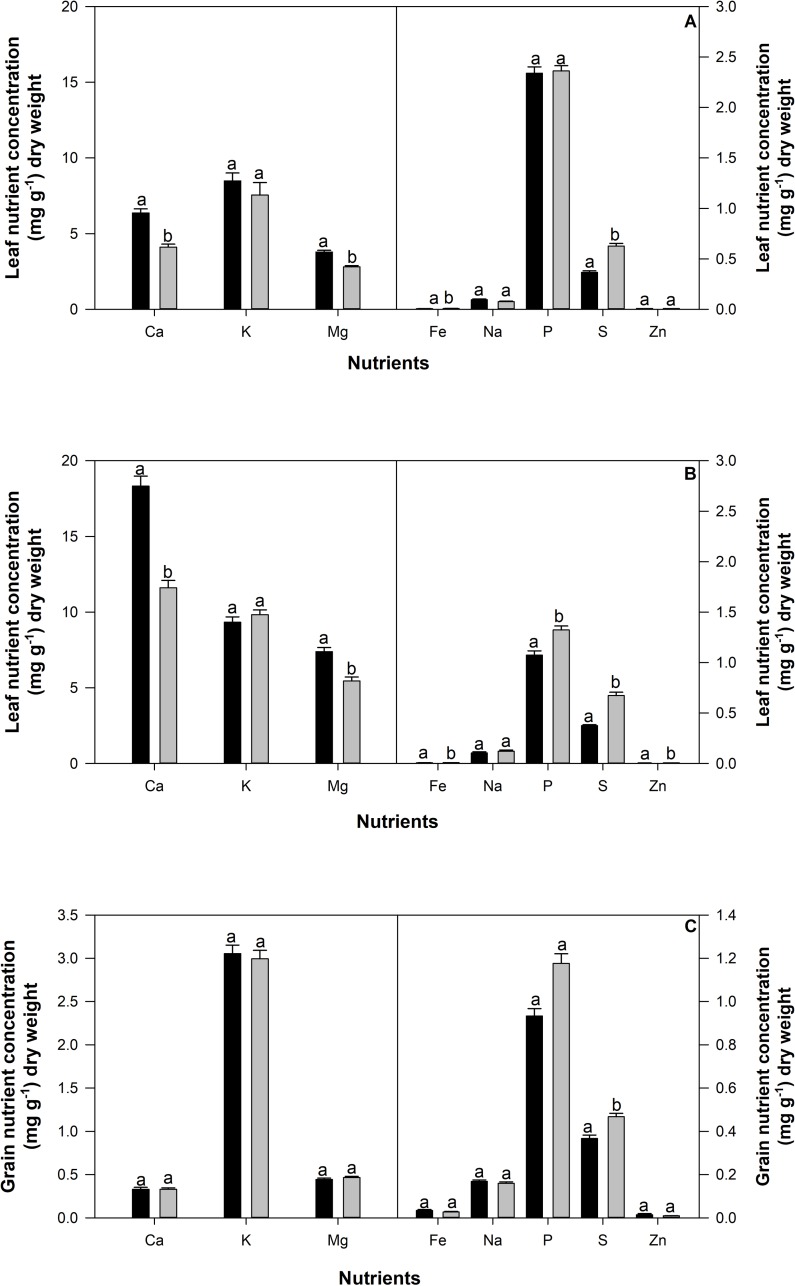
Concentration of nutrients found in the soluble leaf (mg g^-1^) at flowering (panel A) and pod maturity (panel B) and digest seed (panel C) (mg g^-1^) in 12 common bean genotypes grown in the field at CIAT under irrigated (black bars) or rainfed (drought) (grey bars) conditions during flowering (top panel) and pod set (middle panel) and harvest (bottom panel). Letters denote significance between treatments. Statistically significant differences were observed between development stages for soluble leaf nutrients (multiple comparisons are not shown). Standard errors are shown where; n = 36.

**Fig 5:**
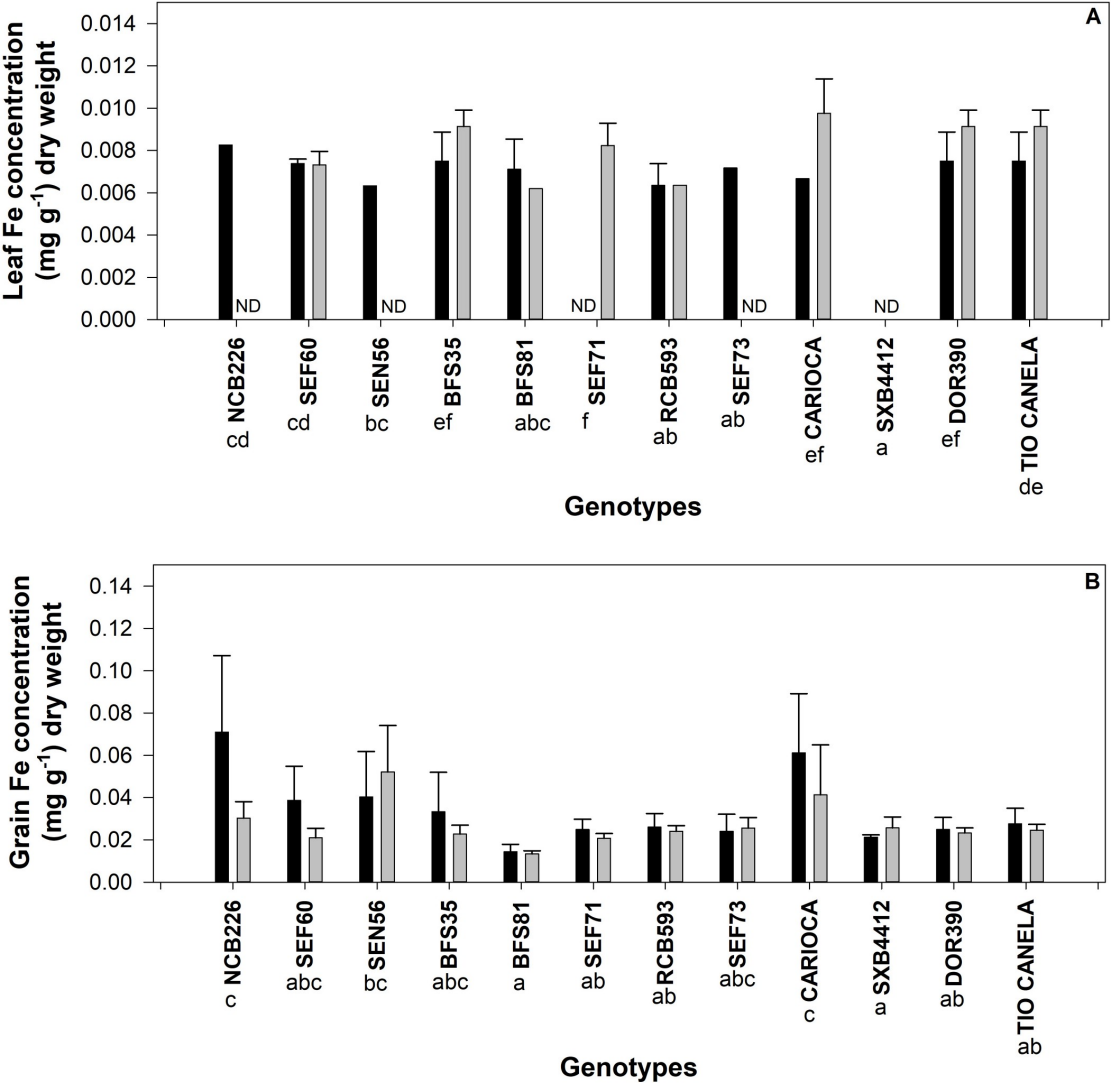
Concentration of iron found in soluble leaf at pod set (panel A) and digest seed at harvest (panel B) (mg g^-1^) found in 12 common bean genotypes grown in the field at CIAT under irrigated (black bars) or rainfed (drought) (grey bars) conditions. Letters denote significance between genotypes. Standard errors are shown where; n = 3.

Variation in response to treatment for calcium, magnesium, iron, sulphur, phosphorus and zinc were observed in the leaf tissue (Fig 4A and 4B). Significant differences in the concentration of all leaf tissue nutrients (except for iron and sulphur) between flowering and pod set were observed (Fig 4A and 4B). An interaction effect between genotype and treatment was observed for iron (Fig 5A), magnesium and sulphur (data not shown). For some genotypes (SEN65 and RCB593) under drought conditions, iron concentration fell below the detection limit of the instrument. In all other genotypes, where detected, concentrations of iron in the soluble leaf fraction in response to drought, were similar to or higher than iron concentrations in irrigated treatments (Fig 5A). For some of these genotypes (NCB226, SEN56, SEF73, Carioca for irrigated treatments and BFS81 and RCB593 for drought treatments), only one sample was identified above the limit of detection, hence no standard error could be calculated. Overall BFS35 and Tio Canela had the highest concentrations of iron in the soluble leaf tissue.

In comparison to the soluble leaf tissue, drought had no discernible impact on the concentration of nutrients within the seed, excluding sulphur which increased in concentration under rainfed conditions (Fig 4C). Statistically significant genotypic differences were detected for iron (Fig 5B) and calcium (data not shown). Iron was detected in all genotypes under both irrigated and drought conditions. For genotypes, NCB226, SEN56, SEF73 and SXB412 which had no iron detected in leaves under drought (or either treatment for SXB412), concentrations of iron in the seed were higher than, or similar to, those detected in the irrigated treatment. NCB226 and Carioca maintained the highest concentrations of iron in the seed under irrigated conditions, while SEN56 and Carioca maintained the highest concentrations of iron under drought.

**Fig 6:**
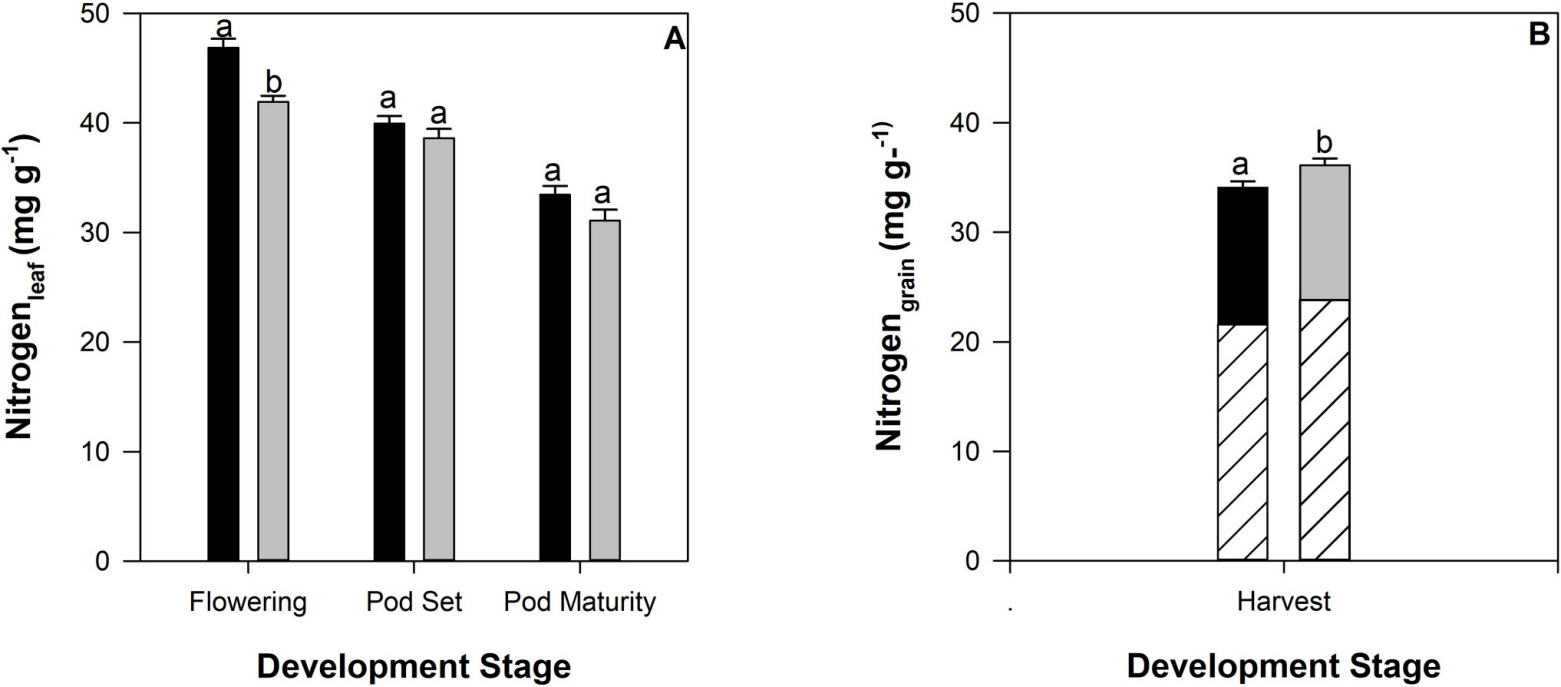
Nitrogen (mg g^-1^) for leaf (panel A) and seed (panel B) tissue from 12 common bean genotypes grown in the field at CIAT under irrigated (black bars) or rainfed (drought) (grey bars) conditions during crop development for leaf tissue; flowering, pod set, and pod maturity. Proportion of amino acids detected in the total grain tissue is displayed in the hashed bars (panel B). Letters denote significance between treatments. Standard errors are shown where; n = 36.

Leaf nitrogen concentration decreased over development (Fig 6A). No significant genotypic differences in leaf nitrogen concentration were detected (data not shown). A slight (0.5%), but significant, increase in seed nitrogen concentration was detected in response to drought conditions (Fig 6B). Genotypic variation in leaf nitrogen was detected within the range of 30–39 mg g^-1^ under both irrigated and drought conditions (data not shown). The proportion of nitrogen in the seed that is accounted for by amino acid concentration was slightly higher for the drought treatment (66%), compared to the irrigated treatment (63%).

### Impact of drought on the concentration of amino acids

**Fig 7:**
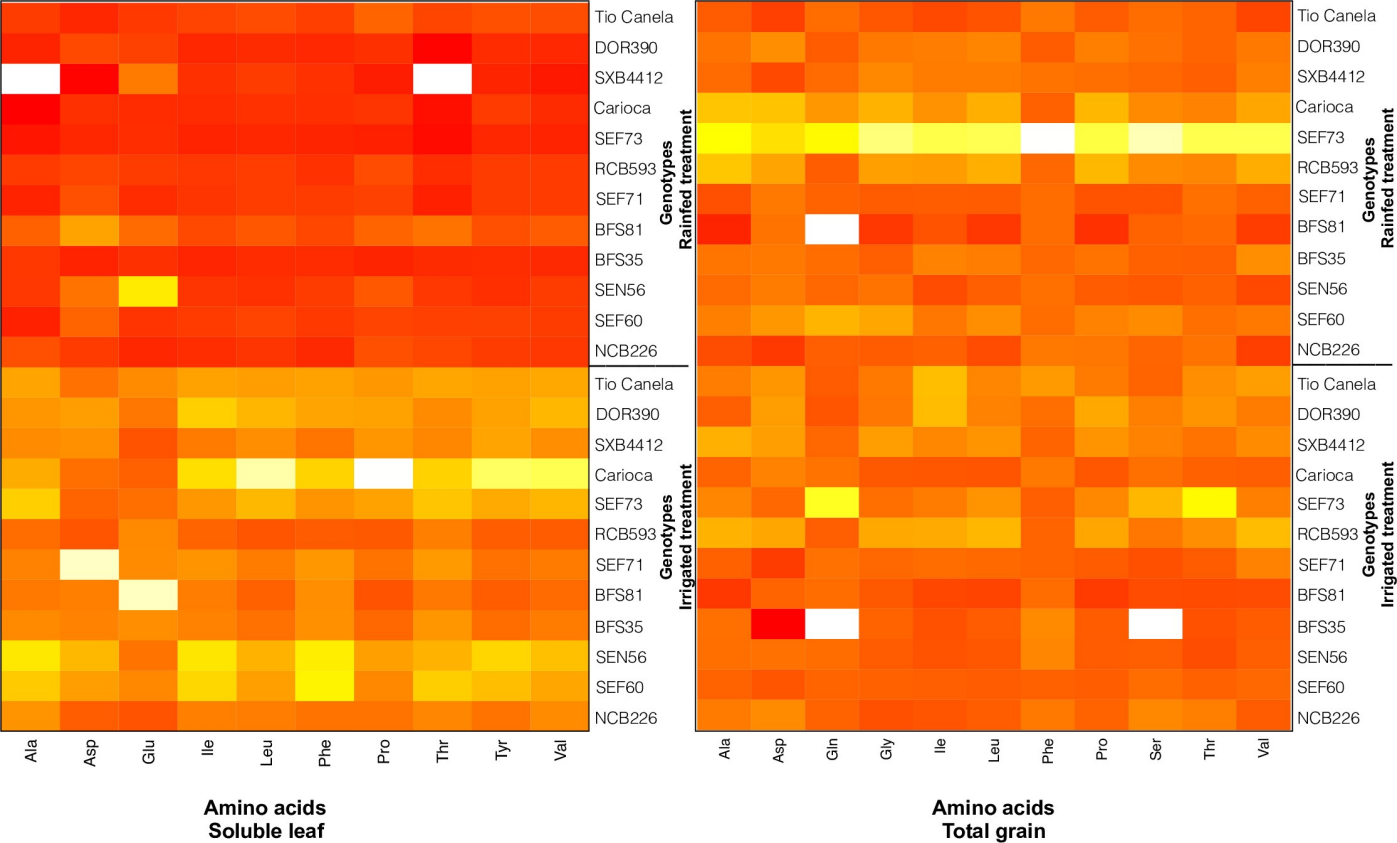
Concentration of amino acids (mg g^-1^ dry weight) found in 12 common bean genotypes (labelled on the left side) of soluble leaf fraction (left panel) and soluble seed fraction (right panel) grown in the field at CIAT under irrigated or rainfed (drought) conditions. Darker colours indicate higher concentrations of amino acids. Blank squares indicate that no amino acid was detected.

Within the soluble fraction of the leaf tissue, statistically significant treatment differences were observed for all amino acids detected (P < 0.01) with a decrease in concentration of these amino acids in response to drought by on average 40% (Fig 7, left panel). A significant interaction between genotype and treatment was observed for all amino acids detected except for glutamine and tryptophan.

In the total seed material, concentrations of amino acids detected under both irrigated and drought treatments did not differ substantially. Under drought conditions significant differences were detected compared to the irrigated treatment for glutamine (average increase of 75%), aspartic acid (average increase of 38%), leucine (average increase of 24%) and glycine (average increase of 22%) (Fig 7, right panel). Significant interactions between genotype and treatment were not detected.

### Impact of drought on sugars

**Fig 8:**
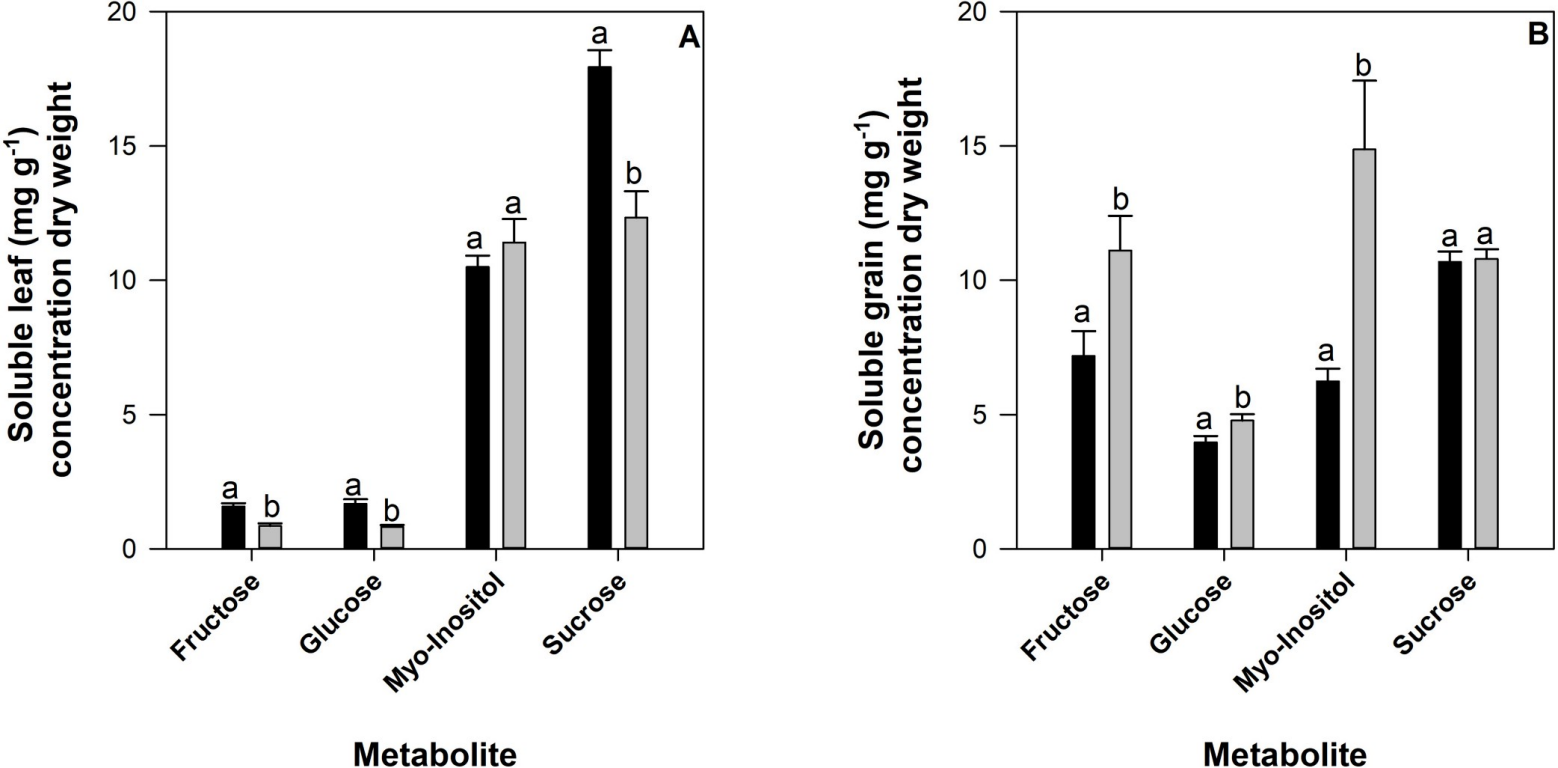
Concentration of sugars (mg g^-1^) found in 12 common bean genotypes grown in the field at CIAT under irrigated (black bars) or rainfed (drought) conditions over development for soluble leaf tissue (panel A) and soluble seed fraction (panel B). Standard errors are shown where; n = 36.

Sucrose accumulated in the highest concentrations within the soluble leaf tissue, followed by myo-inositol with similar concentrations of fructose and glucose (Fig 8A). Statistically significant treatment differences were observed for all sugars found in the soluble leaf tissue (P < 0.001) with the exception of the sugar alcohol, myo-inositol (Fig 8A). Significant differences were also detected for the interaction between genotype and treatment for all metabolites in the soluble leaf tissue (S1 Fig).

Within the soluble fraction of seed tissue, fructose, glucose and myo-inositol increased significantly under drought conditions (P < 0.001) however, no significant change between irrigated or drought conditions was observed for sucrose (Fig 8B). Significant differences were also detected for the interaction between genotype and treatment for all metabolites, excluding fructose, in the soluble seed tissue (S1 Fig).

## Discussion

### Yield quantity reduced under drought conditions

Harvest index was substantially reduced under drought conditions in the field (Fig 1E) with corresponding reductions in whole plant size, reduced leaf area (Fig 1F) and also a likely reduced extent of root exploration. Previous research has suggested that an efficient root system, with greater root vigor and increased rooting depth (see, [19]), is a useful trait in common bean for improved resistance to drought [8, 19]. Overall reductions in plant size under drought conditions corresponded with decreases in seed weight and substantially decreased yield (Fig 1A). As seed weight only accounts for approximately 20% of the four-fold reduction in yield, it is likely that the major impact of terminal drought was on seed number occurring as a consequence of abortion of reproductive tissue in response to reduced resource supply (see, [20, 21]). Declines in pod harvest index (PHI) and pod partitioning index (PPI) suggest that plants experiencing drought were unable to maintain the same rates of remobilization of carbohydrate from vegetative tissue (PPI) and pod wall (PHI) into the seed as was observed with irrigated plants, thereby reducing their capacity to maintain high yields under stress conditions [4, 22–24]. Improved responses by lines that were bred for adaptation to drought stress, in particular, BFS35, NCB226, RCB593 and SEF73 which respond well under both irrigated and drought conditions in the field (Fig 2), suggests that characteristics that promote yield have been retained in breeding programs and enhanced yield resilience under both drought and less challenging conditions (see, [10, 11]).

### Carbon isotope abundance indicated drought stress late in development

Drought did not influence δ^13^C of the leaf tissue, except at pod maturity when δ^13^C of leaves of irrigated plants was lower compared to droughted plants (Fig 3A). A lower δ^13^C suggests a higher leaf internal CO_2_ concentration during photosynthesis, which is expected for well-watered plants. It is not known why δ^13^C also decreased somewhat (though not significantly) in droughted plants compared to earlier stages of crop development. This may be a consequence of changes in stomatal behaviour as the plant develops. It may also be a result of translocation of photoassimilate to heterotrophic tissues increasing as the leaves age, leading to the decrease of δ^13^C in the leaf tissue at pod maturity (Fig 3A) and a higher sink (i.e. seed) δ^13^C at harvest (Fig 3B). This response suggests that plants experiencing drought were able to mitigate the impact by maintaining, or even increasing stomatal conductance until such time as complete stomatal closure was induced [25]. While we didn’t measure stomatal conductance in this study, previous research in common bean suggests that genotypes exhibit a range of stomatal control under drought stress, and that stomatal conductance and yield is positively correlated under both irrigated and drought conditions [19, 26].

### Drought stress disproportionally influenced yield quantity and quality

Nitrogen concentration in the leaf tissue declined over development (Fig 6A) and was not adversely impacted by drought in the seed tissue (Fig 6B). The proportion of nitrogen that can be attributed to total amino acids was determined to be 63% and 66% for irrigated and drought treatments, respectively. The FAO recommends that the assessment of protein content in foods be measured by the sum of individual amino acids, however notes that when such data are not available, it is acceptable to estimate protein content based on total nitrogen content [27]. Using the conversion factor of 6.25 as recommended by the FAO (27), protein content for this study was determined to be 213 mg g^-1^ and 226 mg g^-1^ for irrigated and drought treatments, respectively. This is substantially higher than the protein supplied by total amino acids (Fig 3) suggesting that the conversion factor may be acceptable to estimate crude protein but it may overpredict the amount of available protein within the seed.

Within the soluble leaf fraction at flowering, the concentration of all amino acids detected reduced as a consequence of drought (Fig 7, left panel). These observations did not correspond to what was observed in the total seed fraction where drought impacts were not negative, several amino acids increasing as a consequence of drought (Fig 7, right panel).

For the present study, we were able to compare the amino acids present in the soluble leaf tissue to those in the total grain tissue. The intention was to detect potential markers within the soluble leaf tissue that may be used to rapidly assess nutrient content of the leaf tissue and hence, predict nutrient content of the resulting yield. However, the amino acids found in the soluble fraction of the leaf tissue do not reflect the totality of amino acids present in the total seed tissue nor the quantitative changes in response to drought. Responses of amino acids detected in the total seed tissue support results reported by Gyori, Nemeskeri [28]) that the impact of drought on concentration of amino acids in *P*. *vulgaris* seed is positive overall. Similarly, water stress was found to increase the concentration of amino acids detected in *Cicer arietinum* L. [29].

### Nutrient concentration in plant tissues varied in response to drought stress

Drought had a significant impact on the concentration of the majority of mineral nutrients in the soluble fraction of leaves in this study. Calcium, magnesium, iron, sulphur, phosphorus and zinc significantly decreased by 5–20% under drought (Fig 4A and 4B). Most of these nutrients are relatively immobile in both plant tissues and the soil which may have influenced the capacity of plants to access them from the soil at greater rooting depths [30] or influenced their ability to be remobilized for transport within the phloem, particularly calcium, iron and zinc [31, 32].

In the leaf tissue, δ^13^C did not reflect the impact of drought (Fig 3A). Despite the impact of drought stress being detected via δ^13^C in the seed tissue (Fig 3B), the nutrient concentration found within the total seed tissue did not significantly differ under drought for any of the nutrients except sulphur. The capacity for the seed to buffer the impact of drought and maintain a similar nutrient concentration under both irrigated and drought conditions is possibly a result of the inherent evolutionary requirements of reproductive tissue to enclose an adequate amount of resources for germination of seed. While there is an evolutionary reason for this response, which presumably involves the cessation of flowering, and abortion of non-viable pods and seeds, so that the available nutrients can be distributed to the remaining seeds, the regulatory mechanisms behind the maintenance of nutrient concentration within the seed remain unclear.

Genotypic variation of nutrient concentration in response to drought was detected at the leaf level for magnesium, sulphur and iron, and in the seed for iron and calcium. The capacity to increase the bioavailability of target nutrients via the selection of superior lines is well-established in the literature as a promising mechanism to improve the nutrition of consumers in areas where micronutrient deficiencies are prevalent [33]. While the common bean lines used in this study were not chosen for high iron concentration, the genotypic variability demonstrated in this study in both the leaf and seed tissue (Fig 5) is unsurprising, given that genetic variability for iron concentration in seed of common bean has been reported previously [11, 34]. Iron biofortified beans have recently been released in several African countries [35] with a study in Rwandan women finding significant improvements in the iron status of women who consumed iron biofortified beans [36]. These results suggest that similar improvements in the nutritional quality of common bean can be achieved through breeding activities to exploit genetic variation in the capacity to access and store nutrients under field cropping conditions.

### Drought stress induced significant changes in the concentration of soluble sugars

Soluble carbohydrates fructose, glucose and *myo*-inositol significantly increased in response to drought within the seed in comparison to irrigated treatments. The substantial increase of *myo*-inositol in response to drought is likely a result of the role polyols play in osmoregulation [17, 37]. There is a potential that this significant increase in *myo*-inositol may result in greater amounts of *myo*-inositol-1,2,3,4,5,6-hexakisphosphate (phytic acid) which is the most abundant phosphorylated *myo*-inositol derivative [17, 38]. Phytic acid has a substantial influence on the absorption of iron, zinc, calcium, magnesium and manganese (see, [38]) and is known as a detrimental component of food [39]. Whilst the scope of our investigation focused on a relatively small subset of sugars and sugar alcohols based on their dominance in the soluble fraction, further studies are warranted to investigate the interplay between deleterious photoassimilates, digestion and nutrient availability in diets.

Significant genotypic variation was detected for many of the metabolites measured in this study (Figs 5, 7 and 8). It is challenging to delineate superior genotypes as responses vary depending on the metabolite measured. Nevertheless, this study reinforces that genetic variation in common bean to drought exists not only for yield as previously detected, but also for nutrient concentration, particularly in the leaf tissue. The genotypic variability found for the 12 genotypes used in this study, and genetic diversity detected previously in common bean and close relatives such as *Phaseolus acutifolius*, should, as previously recommended by Beebe, Rao (8), be further researched to identify, and improve, traits that confer drought resistance to common bean in field environments.

Overall, the present study has demonstrated the impact of drought on yield and nutrient concentration in 12 common bean genotypes grown in the field at CIAT. We detected reductions in yield and leaf area, along with genetic variation for these traits. While leaf δ^13^C across crop development, did not reflect the impact of drought, the response of δ^13^C in the seed to drought was significant. Drought significantly influenced the concentration of immobile mineral nutrients and most amino acids detected within the leaf tissue, while not negatively impacting on the concentration of nutrients and amino acids detected within the seed. Increases in amounts of soluble sugars and sugar alcohols should be further investigated for their impacts on digestion and anti-nutritive properties. Genotypic differences were observed demonstrating variation within the gene pool that should be investigated further to identify traits that confer drought resistance.

## Materials and methods

### Experimental site and meteorological conditions

A field trial was conducted from July to October in 2015, at the main experimental station of the International Centre for Tropical Agriculture (CIAT) in Palmira, Colombia, located at 3° 29” N latitude, 76°21” W longitude and an altitude of 965 m. The soil, described previously (see, [10]), is a Mollisol (Aquic Hapludoll) with no major fertility problems. During the duration of the trial, mean maximum and minimum air temperatures were 32.6°C and 19.7°C, respectively, with an average relative humidity of 52%. Rainfall was recorded as 10.1 mm, total potential pan evaporation was 402 mm. Two levels of water supply (irrigated and rainfed) were applied via furrow irrigation with approximately 35 mm of water provided per irrigation event. The rainfed treatment received four irrigations at: 3 days prior to sowing and at 5, 20 and 25 days after sowing. Furrow irrigation was suspended after the application of the fourth irrigation to induce terminal drought stress conditions (reduced water availability from flowering through to physiological maturity). As such, the rainfed treatment will be referred to as the drought treatment throughout the manuscript from this point onwards. The irrigated control treatment received six irrigations over crop development to ensure adequate soil moisture for growth and yield development.

### Plant material and experimental design

Twelve bush bean genotypes belonging to the Middle American gene pool, previously bred by CIAT and its partners, were selected for their inclusion in the trial based on their resilience to abiotic stress or commercial availability (Table 1). Seed color and growth habit of the lines selected varied (Table 1). Growth habits used in this study, as described by Singh [40], are 2A: an indeterminate growth habit lacking climbing ability; 2B: an indeterminate growth habit possessing some climbing ability; and 3B: an indeterminate growth habit with long main stem guide possessing moderate climbing ability. Some of the lines had been characterized previously under drought conditions (see, [9, 24]). A randomized complete block design with three replications was used. Experimental units consisted of four rows with 3.72 m row length with a row-to-row distance of 0.6 m and plant-to-plant spacing of 7 cm (equivalent to 24 plants m^-2^). The field trial was managed with weeding and application of insecticides and fungicides as required.

**Table 1:** Description of 12 common bean lines included in the field trial established at CIAT grown under irrigated and drought conditions. Growth habit classification is explained in the text.

Objective	Line	Seed color	Growth habit
**Superior drought and low P tolerant**	NCB226	Black	2B
	SEF60	Red	2A
	SEN56	Black	2A
	BFS35	Red	2A
	BFS81	Red	2B
**Drought tolerant**	SEF71	Red	2A
	RCB593	Red	2B
**Low P tolerant**	SEF73	Red	2B
	Carioca	Cream striped	3B
	SXB412	Cream	2B
**Commercial checks**	DOR390	Black	2B
	Tio Canela	Red	2A

### Biomass and seed yield measurement

As per the methodology used for previous field trials conducted at CIAT (see, [9]), at mid-pod fill, a 50 cm segment of a row from each plot consisting of approximately seven plants was taken for destructive sampling to measure leaf area index, canopy biomass and dry matter distribution between leaves, stems and pods. At harvest, plants in 50 cm of a row from each plot were cut and dry weights of stem, pod, seed, and pod wall were recorded. To determine seed yield, seed was harvested from the two central rows after discarding end plants in both the irrigated and rainfed plots. Harvest index (HI), pod harvest index (PHI) and pod partitioning index (PPI) were determined by Eqs 1, 2 and 3 respectively as described in Beebe, Rao (8):

$$HI=\frac{seed\;dry\;weight\;at\;harvest}{total\;aboveground\;plant\;dry\;weight\;at\;mid-pod\;fill}\times 100\text{\%}$$ (1)

$$PHI=\frac{seed\;dry\;weight\;at\;harvest}{pod\;dry\;weight\;at\;harvest}\times 100\text{\%}$$ (2)

$$PPI=\;\frac{pod\;dry\;weight\;at\;harvest}{total\;canopy\;dry\;weight\;at\;mid-pod\;fill}\times 100\text{\%}$$ (3)

### Extractions of leaf and seed material

Samples of leaves and seed were microwaved for 10 seconds using a conventional 900W microwave oven to stop metabolic activity according to the method outlined in Popp, Lied [41]. Samples were then oven dried at 85°C and ground using an oscillating matrix mill. Approximately 40 mg of ground sample was then weighed into a 2 mL micro-tube and extracted in hot water according to the protocol outlined in Merchant, Adams [42]. An additional 20 mg of ground seed material was placed with 1 ml of 6M hydrochloric acid in a vacuum hydrolysis tube (Thermo Scientific) and digested for 24 h at 110°C on a heating module (Thermo Scientific, Reacti-Therm III) for protein hydrolysis. Both extracts were stored frozen at -80°C awaiting further analysis.

### Analysis of carbon isotope abundance

Determination of carbon isotope abundance on ground leaf and seed material was completed using a Delta V Advantage isotope ratio mass spectrometer (IRMS) (Thermo Electron) with a Conflo IV interfact (ThermoFisher Scientific, Bremen, Germany). Use of standards maintained the precision of the IRMS at ± 0.1%. Carbon isotope abundance (δ^13^C) expressed as per mil (‰) was calculated as described in [43].

### Analysis of plant material for carbohydrates, amino acids and nutrients

Determination of soluble sugars in extracted samples was completed using an Agilent 7890A gas chromatograph coupled to a triple quadrupole mass spectrometer with a HP5 column (Agilent Technologies, Santa Clara, CA, USA). Derivatisation was completed on lyophilised dried extract which was resuspended in 400 μL of anhydrous pyridine and 50 μL of trimethylchlorosilane (TMCS)/ bis-trimethylsilyl-trifluoroacetamide mix (1:10, Sigma Aldrich, St Louis MO, USA). Split injection was made at 300°C with initial oven temperature of 60°C set for 2 min moving up to 300°C at a rate of 10°C min^-1^ and maintained for 10 min. Column flow rate was maintained at 1.5 ml min^-1^. Peak integration was made using Agilent MassHunter Workstation software (Agilent Technologies, Santa Clara CA, USA).

Determination of amino acids in extracted samples, was completed using high performance liquid chromatography (HPLC) coupled to a quadrupole time-of-flight mass spectrometer. HPLC separation was completed on an Agilent 1290 Infinity system (Agilent, Walbronn, Germany) using a Zorbax StableBond SB-CB18 column (150×2.1 mm, 3.5 μm, Agilent) including degasser, binary pump, temperature-controlled autosampler (maintained at 4°C) and column compartment (maintained at 30°C). The mobile phase was composed of water containing 0.1% formic acid (solution A) and methanol containing 0.1% formic acid (solution B). The flow rate was 0.3 mL min^-1^ with a gradient elution of 0 to 100% solution B, over 23 min for positive mode, respectively. Amino acids were detected using a quadrupole- time-of-flight mass spectrometer (Agilent 6520 accurate-mass) with a dual electrospray ionization (ESI) source. The mass spectrometer was operated with full scan in positive FT mode for amino acid analysis (see, [44]). ESI capillary voltage was set at 4000 V (+) ion mode and 3500 V (−) ion mode and fragmentor at 135 V. The liquid nebulizer was set to 30 psig and the N drying gas was set to a flow rate of 10 L min^-1^. Drying gas temperature was maintained at 300°C. Internal reference ions were used to continuously maintain mass accuracy. Molecular ions ([M+H]+ for amino acids) were extracted from the full scan chromatograms and peak areas integrated using Agilent MassHunter Workstation software (Agilent Technologies, Santa Clara, CA, USA). Limit of reporting was 0.25 mg L^-1^.

Determination of soluble nutrients and total mineral nutrients in extracted and digested samples respectively, was completed using an inductively coupled plasma optical emission spectrometer (Varian Vista, Santa Clara, CA, USA). Samples were prepared with a dilution of 400 μl of supernatant in 10 ml of ultra-pure Milli-Q water. Mineral nutrients: calcium (Ca), iron (Fe), potassium (K), magnesium (Mg), phosphorus (P), sulphur (S) and zinc (Zn), were chosen for analysis. Any results lower than the detection limit of 1 ppb for Ca, Mg, K, Na; 5 ppb for Fe, Zn; and 100 ppb for P, S of the instrument were not taken into account.

### Statistical analysis

Analysis of linear mixed models using the method of residual maximum likelihood (REML) was completed using GenStat 15^th^ Edition (VSN International, Hemel, Hampstead, UK). Fishers unprotected least significant difference (LSD) test was used for post hoc testing. Heatmaps were created using RStudio.

## Supporting information

S1 Fig Concentration of sugars (mg g^-1^) found in 12 common bean genotypes (labelled on left side) of soluble leaf fraction (left panel) and soluble seed fraction (right panel) grown in the field at CIAT subject to irrigated or rainfed conditions. Darker colours indicate higher concentrations of sugars. Blank squares indicate that no amino acid was detected.
(TIF)

S1 File Supplementary information_Smith et al. (XLSX)
