# Supplementary figures and images for: Field drought conditions impact yield but not nutritional quality of the seed in common bean (Phaseolus vulgaris L.)
Source: PLoS One. 2019 Jun 6;14(6):e0217099. doi: 10.1371/journal.pone.0217099 (PMC6553706; doi:10.1371/journal.pone.0217099)

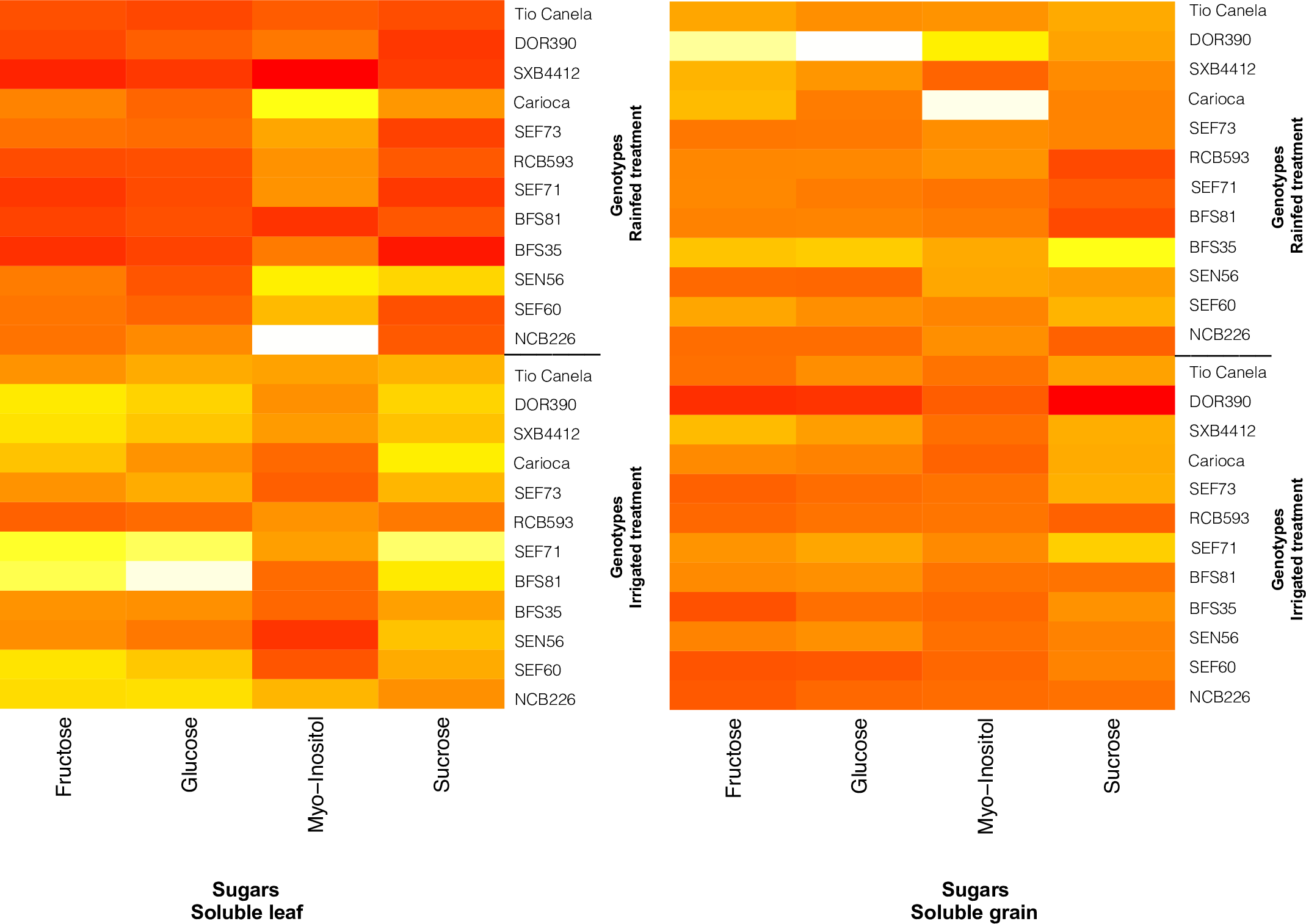

Supplement: S1 Fig — Darker colours indicate higher concentrations of sugars. Blank squares indicate that no amino acid was detected. (TIF) [file pone.0217099.s001.tif]
